# The Sources of Knowledge of the Economic and Social Value in Sport Industry Research: A Co-citation Analysis

**DOI:** 10.3389/fpsyg.2020.629951

**Published:** 2020-12-29

**Authors:** Jose Torres-Pruñonosa, Miquel Angel Plaza-Navas, Francisco Díez-Martín, Camilo Prado-Roman

**Affiliations:** ^1^Facultad de Empresa y Comunicación, Universidad Internacional de La Rioja, Logroño, Spain; ^2^Institución Milá y Fontanals de Investigación en Humanidades, Consejo Superior de Investigaciones Científicas, Barcelona, Spain; ^3^Department of Business Economics, Universidad Rey Juan Carlos, Madrid, Spain

**Keywords:** economic value, social value, sports, intellectual structure, bibliometric, trends, co-citation, multi-fiduciary theory of stakeholders

## Abstract

The aim of this article is to map the intellectual structure of scholarship on economic and social value in the sport industry. Given that bibliometric techniques are specially appropriate for identifying the intellectual structures of a field of knowledge and complement traditional literature reviews, a co-citation bibliometric analysis has been applied. This kind of analysis identifies networks of interconnections. Therefore, we aim to detect both the most and the least active research areas in this field, as well as their sub-disciplinary composition. There is an abundance of literature on sport efficiency and economic efficiency in the sport industry, our main conclusion is the identification of a literature gap in regard to social value in sport organisations, which is expected to be a research opportunity for scholars. This is in line with the lack of standardisation in the measurement for social value in sport organisations. In fact, similar to analysis undertaken in the past few decades of other industries with contributions to stakeholders and the multi-fiduciary theory of stakeholders, both the creation of social value indicators for sport entities and the empirical analysis of social efficiency in sport institutions, are identified and outlined as future areas of research. Therefore, this bibliometric analysis will contribute to determine the future challenges that this area of research will face in the following years so as to fill the literature gap identified.

## Introduction

Companies have been considered, since the inception of the industrial age, as entities that generate economic value (Groth et al., 1996). Therefore, their social role has been forgotten or relegated to a second stage (Anunciaçao et al., 2011; Retolaza et al., 2015). For this reason, many sophisticated quantitative methodologies have been developed to assess the economic value and, consequently, reflect the faithful image of a company by means of financial reports (Gassenheimer et al., 1998).

Nonetheless, over the last few decades, some scholars (Jensen, 2001; Argandoña, 2011) have proposed an integrated paradigm, given that companies are not only generators of economic value, but also of social value. In spite of the fact that initially the approach was taken under a subtractive viewpoint (by means of negative externalities), thereafter, a more positive perspective was taken by means of the development of concepts like corporate citizenship (Maignan et al., 1999; Maignan and Ferrell, 2001; Néron and Norman, 2008) and Corporate Social Responsibility (Carroll, 1999). Nevertheless, only a small amount of research initially incorporated this evolved perspective in regard to economic and social value generation (Nelson and Winter, 1982; Williamson and Winter, 1993). It is true that different methodologies (Olsen and Galimidi, 2008; Tuan, 2008; Mulgan, 2010), such as Social Return on Investment (SROI) (Lingane and Olsen, 2004), social accounting (Lazcano et al., 2019; Retolaza et al., 2020) or social value monetisation (Retolaza et al., 2016) have emerged in order to provide quantitative methodologies. Nonetheless, even though there has been an increasing concern among scholars to assess the social value of companies, there is still the need to cover the gap relative to quantitative methodologies that will allow for the measurement of the social value that enterprises generate.

This is the case with the sport industry, were theoretical and qualitative studies on Corporate Social Responsibility and emotional value are common (i.e., Smith and Westerbeek, 2007; Godfrey, 2009; Lock et al., 2012), but quantitative studies are few. This does not mean that this field of knowledge is decreasing. In fact, only 3.4 papers were published per year during the period 2001–2005 in the field of economic and social value in the sport industry. This amount has dramatically increased over the last few years: 15 papers per year during 2006–2010, 25 in 2011–2015, and 55.2 in 2016–2020. In fact, this increasing number of publications on economic and social value in the sport industry makes it complicated to track this developing research area.

As a matter of fact, when knowledge increases in any field, it must some how be analysed. Take the example of the shared value concept developed by Porter and Kramer (2011) which takes into account both the economic and social advancing effects of policies and operating practices that enhance the competitiveness of companies. Many literature reviews have been recently developed upon this concept under different perspectives and with both qualitative (Voltan et al., 2017; Laudal, 2018) and quantitative approaches (Uribe et al., 2018; Maestre-Matos et al., 2020). Nonetheless, no one makes a segmentation using sports as a criterion.

The main objective of this article is to determine and provide a vision of the intellectual structure and dynamics of economic and social value in the sport industry field. This implies delimiting the scientific domain’s research traditions, their disciplinary composition, and influential research topics (Shafique, 2013). As opposed to classical reviews that only increase the conceptual understanding of a field of knowledge, this article contributes to the field by showing its intellectual structure. Additionally, we provide researchers with a basis for the future development of this field of study by means of the identification of the main areas of research, the main contributions that have led to its circulation, which have captivated the special attention of researchers, the trends that have taken place, and the proposal of future lines of research.

When it comes to sport industry literature reviews that deal with economic and social value, the majority of papers have taken a qualitative approach. Bayle (2016) reviews Olympic social responsibility literature. Bjärsholm (2017) reviews social entrepreneurship in sports. Giulianotti (2015) critically reviews CSR in sports. With regard to valuation methods, whereas Orlowski and Wicker (2019) perform a critical analysis of monetary valuation research in sports, Keane et al. (2019) critically analyse the methods used to quantify the social and economic value of active recreation and sports. Walzel et al. (2018) critically review the literature on professional team sports organisations’ CSR and conclude that the approach is mostly qualitative. Reviews that deal with economic and social value in the sport industry take either a quantitative approach or undertake a systematic review. For instance, Kim (2017) performs a quantitative meta-analytic review of Korean sport literature with regard to CSR practices, whereas Mallen et al. (2011) perform a content analysis of environmental sustainability research published in sport-related journals.

The most important contribution of these reviews is that they set the theoretical and conceptual framework. Consequently, they are essential for developing the research field. Nonetheless, some items have not been identified in spite of being key aspects in comprehending how the research field has evolved as well as its current situation. These following questions remain, therefore, unanswered: What are the main current areas of research on economic and social value in the sport industry? What papers have allowed the dissemination of the field of knowledge? What papers have set the research trends, producing a larger attraction of scholars, and when did that happen? In other words, the intellectual structure of this field of research needs to be defined. This would help scholars to detect the objectives of future research, to carry out research that contributes to current research areas and to include new research areas into this field of knowledge.

Methodological bibliometric analysis allows for the evaluation of huge amounts of data from thousands of academic publications. It offers an evaluation of quantitative data and infers qualitative aspects. Nonetheless, citation or co-citation analysis must be supported by good knowledge of the field of study (Wallin, 2005). Nevertheless, if bibliometric analysis is carried out properly, it is possible to achieve a good view of the subject studied, its clustering and their importance, intellectual turning points, the detection of bursts in influential papers, and research gaps to cover, and so forth.

All things considered, there are two main ways to evaluate academic research: qualitative and quantitative. The qualitative approach, among other aspects, refers to peer-review and the quantitative one, to bibliometric analysis. Currently, due to their great degree of subjectivity, qualitative methodologies are not the best for the recognition of the intellectual structure of a field of knowledge. In this regard, an excess of new, but relevant papers can easily saturate researchers’ information-processing capabilities. Furthermore, the results could be intrinsically biased because authors tend to mirror their subjective viewpoints (Ramos-Rodríguez and Ruiz-Navarro, 2004; Ball, 2018). Therefore, we have opted for a quantitative approach and we apply a citation-based bibliometric methodology to build approximations of research activity. Despite this and other limitations, it is true that bibliometric analysis attempts have been made in order to infer qualitative aspects (Ball, 2018).

This article has the following structure. The methodology is analysed in section ‘Methodology’. The results of the bibliometric analysis are shown in section ‘Results’, namely the cluster definitions, the intellectual turning points, and the most active areas of research. Finally, both the discussion of the implications of these findings and futures lines of research are suggested in section ‘Discussion and Conclusion’.

## Methodology

A bibliometric study of papers on economic and financial value in the sport industry has been carried out so as to obtain insight into its intellectual structure. As a matter of fact, bibliometric methods help academia to comprehend the inception and growth of a field of knowledge. Additionally, bibliometrics are a good complement to traditional literature review (Ramos-Rodríguez and Ruiz-Navarro, 2004). Bibliometric methodology aims to examine the publication performance of researchers and to disclose the structure and dynamics of science (Zupic and Èater, 2015), assisting in the discovery of old and current fields of research, as well as intuiting new ones and possible gaps (Zhao and Strotmann, 2015). Many bibliometric methods have been used in regard to sports research. A search in the Social Science Citation Index (SSCI) shows in the region of 130 papers that deal with bibliometrics in sports. These are some examples: emotions and sport management (Baier-Fuentes et al., 2020), biomechanics (Knudson, 2020), handball (Pardo Ibáñez et al., 2020), sport publications (Phillips, 2020), sexual margination in schools (Sáenz-Macana and Devís-Devís, 2020), sustainable entrepreneurship (Calabuig-Moreno et al., 2020; Escamilla-Fajardo et al., 2020; González-Serrano et al., 2020; Pellegrini et al., 2020), talent evaluation (Zheng and Liu, 2020), sports tourism (Jiménez-García et al., 2020), collaboration and productivity patterns (Pérez-Gutiérrez and Cobo-Corrales, 2020), motivation measures (Clancy et al., 2017) and brain injury (Sharma and Lawrence, 2014), among others.

Having said that, among the possible types of bibliometric and citation analysis, co-citation analysis techniques have been used in this article, given that this kind of analysis is one of the most highly validated and used (Zhao and Strotmann, 2015; Zupic and Èater, 2015). As is well-known, citation analysis is based on the fact that a citation represents some kind of interest in the cited reference from the author who makes the citation; and, a citation shows some relation between cited works and the citing work. Small (1973) defines co-citation as the frequency that two works are cited together. Therefore, two papers are co-cited if they are included in the same work. In actual fact, similarity in publications is assessed depending on the amount of overlap in their bibliographic references, assuming that co-cited papers will have related content. Furthermore, co-citation analyses offer a method for sorting the outstanding papers in a scientific area. In spite of the fact that the recount of cites measures the relative influence of a document, co-cites analysis detects interconnections among papers, identifies networks, and reveals changes in paradigms and lines of thought (Zupic and Èater, 2015). Therefore, the analysis of co-cites is able to map the intellectual structure of a field of research, detect research area trends, discover front-line studies, and highlight discoveries with high impact (Zhao and Strotmann, 2015).

The use of bibliometric maps to represent how different kinds of objects of study (authors, papers, journals, organisations, etc.) are related to one another are considered a useful way to help its visualisation and its comprehension (Cobo et al., 2011). Many software programs are currently used (Moral-Muñoz et al., 2020) in order to perform citation and co-citation analyses via bibliometric mapping, namely: CiteSpace, VOSviewer (van Eck and Waltman, 2010), SciMAT (Cobo et al., 2012), CitNetExplorer (van Eck and Waltman, 2014), BibExcel (Persson et al., 2009), Sci2Tool (Sci2 Team, 2009) and Bibliometrix (Aria and Cuccurullo, 2017), among others. Each software programme has its advantages and disadvantages (Cobo et al., 2011). For instance, Bibliometrix is written in the R language which allows for the interconnection of multivariate analysis packages written in the same language, such as FactoMineR (Lê et al., 2008). Different kinds of analysis can be performed from mapping similar networks, temporal geospatial analysis or burst detection (Cobo et al., 2011), or a combination of all or part of them to obtain not only quantitative measures but also to infer qualitative results.

In this regard, CiteSpace (Chen et al., 2010) has been used in this article, given that it has previously been applied in the business research field in order to analyse the intellectual structure of different areas (e.g., Seyedghorban et al., 2016; Díez-Martín et al., 2020) and because it has some advantages in comparison with the other software options, such as burst detection to identify the main incipient research trends in a field of knowledge, the identification of the foremost turning points and growing topics, and so forth.

### Data

Many bibliographic databases focus on business or economics (ABI/Inform Global, EconLit, Business Source Complete, etc.) and on sports (SportDiscus, Rehabilitation & Sports Medicine Source, Sports Market Analytics, etc.). But given that the SSCI is one of the most frequently used databases to conduct bibliometric analysis in both business (Zupic and Èater, 2015) and sports^1^, all the scientific journals included in the SSCI database have been selected in order to conduct the study. The following Boolean search of terms in the title, abstract, or keywords (TS) have been used: TS = sport^∗^ AND (TS = ‘soci^∗^ valu^∗^’ OR TS = ‘soci^∗^ effi^∗^’ OR TS = ‘soci^∗^ performanc^∗^’ OR TS = ‘soci^∗^ responsib^∗^’ OR TS = ‘soci^∗^ accounting’ OR TS = ‘economic^∗^ valu^∗^’ OR TS = ‘economic^∗^ effi^∗^’ OR TS = ‘economic^∗^ performanc^∗^’ OR TS = ‘financ^∗^ valu^∗^’ OR TS = ‘financ^∗^ effi^∗^’ OR TS = ‘financ^∗^ performanc^∗^’). In other words, a combination of economic^∗^, soci^∗^, or finance^∗^ with valu^∗^, effi^∗^, or performanc^∗^ has been used along with ‘soci^∗^ accounting’ and ‘soci^∗^ responsib^∗^’ for sport literature. As a matter of fact, 90 more articles were found when the same research terms were entered including both the SSCI and Science Citation Index Expanded (SCI-Expanded) databases. Nonetheless, only 10 of them are included in the Sport Sciences Research Area and the Web of Science (WOS) Category. Therefore, it was decided that the SCI-Expanded database would not be included in the analysis given that 80 papers did not deal directly with sports.

The time frame for the study (2000- November 2, 2020) and the thematic category parameters produced 494 citing papers containing 22,607 different cited references, which comprised the data sample of the analysis. The 494 citing publications are included in 201 journals related to these areas. Of those, some are multidisciplinary while others are highly specialised. Table 1 displays the number of articles on economic and social value in the sport industry published by the journals that have four or more published papers, as well as some variables of the Key Indicator 2019 of Journal Citation Reports, namely Journal Impact Factor, 5 Year Impact Factor, and the highest quartile of any of WOS categories in SSCI.

**TABLE 1:** Top 26 journals that have published research citing articles related to economic and social value in the sport industry.

**Journals**	**No. articles**	Journal impact factor*	5 years impact factor*	Quartile*
European Sport Management Quarterly	39	1.889	2.436	Q3
Journal of Sport Management	34	2.359	2.877	Q2
Sport Management Review	29	3.337	3,761	Q2
Journal of Teaching in Physical Education	18	1.845	2.490	Q2
International Journal of Sports Marketing Sponsorship	17	1.075	1.217	Q4
Sport in Society	16	0.939	n/a	Q3
Journal of Business Ethics	11	4.141	5.453	Q1
Sustainability	10	2.576	2.798	Q2
Journal of Management Organization	9	1.935	1.808	Q3
Physical Education and Sport Pedagogy	9	2.618	3.091	Q1
Movimento	8	0.365	0.465	Q4
Journal of Business Research	7	4.874	5.484	Q1
Sport Education and Society	7	2.649	2.892	Q1
Sport Marketing Quarterly	7	0.744	1.226	Q4
International Journal of the History of Sport	6	0.277	0.503	Q4
Journal of Sport Social Issues	6	1.939	1.953	Q2
European Physical Education Review	5	2.393	2.748	Q1
International Journal of Environmental Research and Public Health	5	2.849	3.127	Q1
Management Decision	5	2.723	2.886	Q2
Public Relations Review	5	2.321	2.232	Q2
Quest	5	2.844	2.560	Q1
Revista de Psicologia del Deporte	5	0.677	1.042	Q4
International Review for the Sociology of Sport	4	2.019	1.972	Q2
Leisure Studies	4	1.566	2.349	Q3
PLOS One	4	2.740	3.227	Q2
Urban Forestry Urban Greening	4	4.021	4.468	Q1

*According to key indicator 2019 of journal citation reports.

From the first overview of the 494 citing publications, it is observed that more than 94.13% of the documents retrieved are articles and that 4.45% are reviews with a minimum presence of editorial material, book reviews, and meeting abstracts. Among the 34 institutions with seven or more publications in this area, there are 20 from United States universities, three from Australia and Spain, two from Canada and England and one from Germany, South Korea, Norway and Belgium. Table 2 shows the countries that publish more in this field. The United States is the country with the highest number of publications (188) which represents in the region of 33.75% of the total amount of citing papers published in the sport field. This is less than the percentage of papers written by United States authors in SSCI (40.25%) from 2000 to 2020 (which corresponds to the timespan of the analysis). On the other hand, countries that publish more on economic and social value in the sport industry in comparison with the total amount of papers published in SSCI are Spain (8.62 against 2.92%) and Australia (10.59 against 5.77%). The most prolific authors are P.M. Wright with 13 publications, K. Babiak with 11 and C. Anagnostopoulos, Y. Inoue, and A. Willem with 10 publications each one. Table 3 shows the language used to write the citing papers included in the database. The most used language to publish in this area is English (472 publications), followed at a long distance by Spanish (13), Portuguese (4), French, German, Lithuanian, Russian, and Turkish with 1 publication each. Whereas the percentage of citing papers is similar in English in comparison with the percentage of papers published in this language in SSCI from 2000 to 2020, there are some differences in some languages. For instance, German represents 1.43% of all the published material in SSCI but only 0.20% of the papers of the database analysed have been written in this language. On the contrary, Spanish which represents 0.99% of SSCI publications, represents 2.63% of the published citing papers on economic and social value in the sport industry. To end this descriptive quantitative overview of WOS, the categories with more publications are: hospitality, leisure, sport & tourism (197), management (108), sport sciences (75), business (68), education & educational research (67), sociology (38), environmental studies (29), environmental sciences (23), social sciences interdisciplinary (22), economics (18) and psychology applied (18). Had we used WOS areas of research, the top positions would include: social sciences other topics (235), business economics (172), sport sciences (75), education & educational research (68), psychology (41), environmental sciences ecology (40) and sociology (38).

**TABLE 2:** Top countries with authors that have published citing papers related to economic and social value in the sport industry in comparison with SSCI.

**Countries**	**Sport papers**	**% sport**	**% SSCI**
United States	188	33.75	40.35
England	55	9.87	10.91
Canada	42	7.54	5.78
Australia	59	10.59	5.77
Germany	19	3.41	5.08
People’s Republic of China	16	2.87	4.17
Spain	48	8.62	2.92
Italy	12	2.15	2.45
France	22	3.95	2.44
Sweden	10	1.80	1.86
Scotland	10	1.80	1.34
Belgium	14	2.51	1.29
South Korea	18	3.23	1.19
Taiwan	13	2.33	1.12
Norway	16	2.87	1.10
New Zealand	15	2.69	0.91

**TABLE 3:** Language of citing papers related to economic and social value in the sport industry in comparison with SSCI.

**Languages**	**Sport papers**	**% sport**	**% SSCI**
English	472	95.55	95.81
German	1	0.20	1.43
Spanish	13	2.63	0.99
French	1	0.20	0.59
Portuguese	4	0.81	0.35
Russian	1	0.20	0.23
Turkish	1	0.20	0.07
Lithuanian	1	0.20	0.01

The bibliometric analysis carried out has included all the 22,607 cited references of the 494 citing documents, in other words, the sources of knowledge of economic and social value in the sport industry. This is not to say that only articles of journals have been analysed. As a matter of fact, non-journal articles, (i.e., books) have also been included, given that the analysis is focussed on the co-cited references of the 494 citing papers. In other words, the clustering is conducted with the 22,607 cited papers.

Table 4 shows the selected parameters used to run the analysis through CiteSpace (Timelice, Term source, Node type, Pruning and Selection criteria). The identification of clusters in this mapping software needs to be guided by a careful definition of which source of terms must be used. In order to obtain a source of terms as complete as possible, we opted, in our study, for the broadest option in SSCI with the inclusion of title, abstract, and keywords. Although this option allows us to obtain a good range of terms to be analysed from the 494 citing papers located in SSCI, CiteSpace can improve and complete the analysis by also including the terms that appear in the 22,607 references cited in them [named in the program: ‘keywords plus (all)’]. It is evident that this procedure guarantees a fairly broad source of terms which are well related to the field of study to be analysed.

**TABLE 4:** Parameters for the analysis.

**Parameter**	**Description**	**Choice**
(1) Timeslice	Timespan of the analysis	From 2000 to 2020
(2) Term source	Textual fields processed	Title/abstract/author keywords/keywords plus (all)
(3) Node type	The type of network selected for the analysis	Cited reference (the networks are made up of co-cited references)
(4) Pruning	It is the process to remove excessive links systematically	None
(5) Selection criteria	The way to sample records to form the final networks	g-index (*k* = 25). The g index is the largest number that equals the average number of citations of the most highly cited g publications. It solves some of the weaknesses of the h-index. *k* is a scaling factor introduced in CiteSpace to control the overall size and clarity of the resultant network

The node selection criteria are an important decision in order to achieve a well cohesive network where clusters are different from each other, while containing similar papers within them. CiteSpace offers several node selection criteria: g-index, Top *N*, Top *N*%, and Threshold Interpolation. We based our choice of the g-index as a node selection criteria which enables the generation of a network with the least number of small clusters and, therefore, a better visualisation of nodes and links (Chen and Song, 2019). The g-index measures the global citation performance of a set of articles (Egghe, 2006) and improves the limitations of the h-index because it gives more weight to the highly cited articles and there is no influence due to the total number of considered works (Costas and Bordons, 2008). A scaling factor *k* to the g-index is introduced in CiteSpace in order to regulate the overall size of the obtained network. To develop the most appropriate network for our set of analysed terms we opted for *k* = 25. The obtained structural network quality following the silhouette and modularity measurements are shown in section ‘Results’.

## Results

**FIGURE 1:**
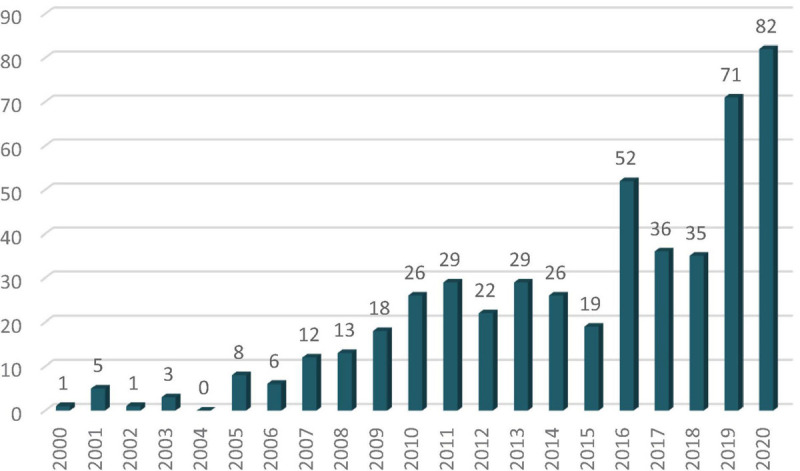
Growth of citing publications on economic and social value in sport industry research (2000–2020).

Research in the field of economic and social value in the sport industry has had, as a whole, a clear growth in the last two decades, with a pronounced increase over the last 2 or 3 years (Figure 1). The growth was slight between 2000 and 2006 (from one to six publications per year), with a slight increase between 2007 and 2015 (with an average of around 20 publications per year) and a clear upswing from 2016 to the present (between 35 and 82 publications per year). This research production has been mainly published in ‘hospitality, leisure, sport & tourism’, ‘management’, ‘sport sciences’, ‘business’, ‘sociology’, ‘environmental studies & sciences’, ‘social sciences’ and ‘economic’ journals. If the total of 494 citing articles published in the study period (2000–2020) is taken into account, an average of 23.5 articles per year is obtained, which is not a very high figure. However, the fact that, the articles in this field of study have been included in high-impact journals is an indication that a certain field of knowledge has been developing in the field of social and economic value in the sport industry. Up to what point? This is what we intend to identify in this section.

### Main Research Areas in Economic and Social Value in the Sport Industry

Table 5 shows the main research areas of economic and social value in the sport industry. The network is split into 10 main co-citation clusters (from #1 to #10), where each one of them corresponds to a different thematic structure. The results (see Supplementary Material) obtained in the form of clustering show the impact of subsequent research co-citing. Take the example of cluster #1, that is formed of 69 cited papers. This means that many subsequent researchers have used several of these 69 cited papers together as a source of knowledge. Therefore, cluster #1 is considered as a thematic structure. Despite the fact that former researchers might have thought that their papers were a contribution to one thematic structure, their research has eventually been used in alternative ways, which may create new research avenues and, consequently, new clusters. All in all, every single cluster relates to a thematic structure or line of research.

**TABLE 5:** Main research areas in economic and social value in the sport industry.

**Cluster**	**Size**	**Silhouette**	**Mean (year)**	**Label**	**Description**
1	69	0.958	2009	CSR theoretical tramework	The concept of CSR in the sport industry is defined and its determinants, drivers, and influential factors are explored
2	54	0.947	2014	CSR case studies	Implementation and decision-making of CSR in the sport industry are analysed by means of case studies
3	45	1.000	2016	Teaching, personal, and social responsibility (TPSR)	The TPSR model is analysed
4	40	0.925	2007	Strategic CSR	Both the general strategic approach of CSR and for the football industry are established
5	37	0.988	2016	Sponsorship	The effectiveness factors of sponsorship, its function as a communication strategy, and the roles of sponsors on recipients are analysed
6	36	0.909	2012	Emotional value	Contributions to social identity theory applied in the sport industry in regard to brand identity, team identification, and attachment
7	19	0.991	2012	Educative programs	Social impact of sport-based programs is analysed
8	18	0.963	2007	Consumer ethical perception	The effect of CSR efforts according to the ethical motives perceived by customers is analysed
9	9	0.983	2008	Environmental issues	Different issues regarding environment are analysed
10	6	0.996	2009	Corporate citizenship	It deals with the specific activities that a sport organisation engages in to meet their social obligations

*Silhouette: quality of a clustering configuration (Rousseeuw, 1987), suggested parameters between 0.7 and 1 (Chen et al., 2010).*

The criterion used to select the 10 clusters was the cluster silhouette value, which must be between the range of 0.7 and 1.0 according to Chen et al. (2010). This value assesses the quality of a clustering configuration by means of cohesion and separation. Cohesion refers to how similar an object is compared to its cluster. Separation has to do with how similar an object is to its own cluster in comparison with other clusters.

Additionally, modularity Q assesses the quality of the overall network division. According to Newman’s method, the value ranges from 0 to 1 (Newman, 2006). High values imply that the clusters created have clear boundaries. Conversely, low modularity values suggest a bad-structured network (Chen et al., 2009).

**FIGURE 2:**
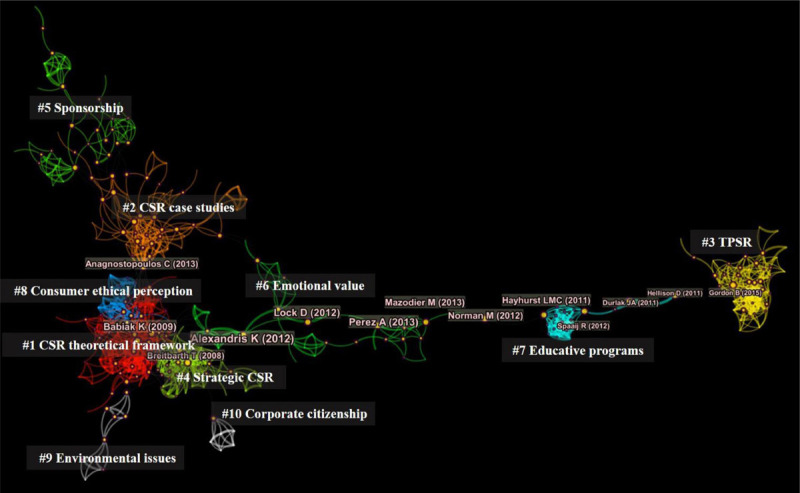
Economic and social value in the sport industry network.

All 10 major clusters that have been created have silhouette values higher than 0.9 which mean that there is good homogeneity between clusters. On the other hand, the modularity Q value is 0.8734, which means that the network that has been created is divided reasonably into loosely coupled clusters. Figure 2 shows the economic and social value in the sport industry network.

Nonetheless, the main limitation of co-citation analysis techniques is that both the interpretation of clusters and the identification of the core thematic structure is based on the domain knowledge and experience of the analysts. Therefore, evidence-based findings are difficult to be distinguished from heuristics and speculations (Zupic and Èater, 2015). In order to both allow for the summation of the essence of each cluster and enhance the robustness of the co-citation cluster labelling process, the common links between the researchers of each cluster as well as citations have been analysed.

Cluster #1 has the biggest number of papers. Therefore, this is the largest research area. This cluster encapsulates the **CSR Theoretical Framework** in the sport industry. This research area introduces and defines the concept of CSR in the sport industry (Godfrey, 2009; Sheth and Babiak, 2010), explores its social role (Smith and Westerbeek, 2007; Walker and Kent, 2009), and identifies its determinants, drivers and influential factors (Babiak and Wolfe, 2009; Sheth and Babiak, 2010; Babiak and Trendafilova, 2011).

The second largest cluster (#2) includes **CSR Case Studies** in the sport industry in regard to the decision making process (Anagnostopoulos and Shilbury, 2013; Anagnostopoulos et al., 2014) and its implementation (Anagnostopoulos and Shilbury, 2013; Heinze et al., 2014). Some of the cases analysed are the Foundations of English football clubs (Anagnostopoulos and Shilbury, 2013; Anagnostopoulos et al., 2014), London 2012 Olympic Games (Dowling et al., 2013) and the Detroit Lions (Heinze et al., 2014).

Cluster #3 has the highest possible silhouette value (1), meaning that their papers have both cohesion among them and separation in comparison with the rest of the clusters. This cluster, along with #5, is among the most rapidly advancing given that their mean publication year is the latest. The **Teaching, Personal and Social Responsibility (TPSR)** model (Hellison, 2003, 2011) constitutes the main research topic of this cluster which is the third largest. This model is based on the concept that teachers can promote personal and social responsibility by means of sport and physical activity. TPSR has five goals or levels of progression in regard to personal and social responsibility: (1) self-control, (2) participation, (3) self-direction, (4) caring, and (5) transfer of responsibility to other settings (Gordon and Doyle, 2015; Hemphill et al., 2015; Gordon et al., 2016; Pozo et al., 2018).

The fourth largest cluster is the one investigating **Strategic CSR** (cluster #4). In this regard, whereas Porter and Kramer (2006) approach CSR strategically without segmenting by kinds of business, Breitbarth and Harris (2008) develop a conceptual model to apply CSR in the football industry in order to achieve better strategic direction.

The fifth most important cluster is **Sponsorship** (#5). Chronologically, whereas Kim et al. (2015) analyse the influential factors to make sponsorship effective, Pappu and Cornwell (2014) consider the roles of both sponsors and recipients. Bason and Anagnostopoulos (2015) conclude that, to all intents and purposes, sport sponsorship represents a business transaction instead of a philanthropic action. For this reason, the former have not been affected during recession years whereas the latter have decreased. Bergkvist and Taylor (2016) consider sponsorship to be one out of five different leverage marketing communications strategies and analyse its effect on the brand. Woisetschläger et al. (2017) examine how sport sponsorship affects consumers’ attitudes and intentions. Finally, according to Zeimers et al. (2019), sponsors in sports are considered as established external stakeholders that are essential sources of organisational learning.

Cluster #6 deals with **Emotional Value**, encapsulating brand identity and loyalty (Pérez et al., 2013) with different contributions to social identity theory (Tajfel and Turner, 1986) applied in the sport industry. Lock et al. (2012) integrate this theory in order to explore how team identification develops in relation to a new sport team. Alexandris and Tsiotsou (2012) segment football spectators according to their degree of attachment to the team, using self-expression and team involvement variables.

Cluster #7 covers **Educative Programs** as its area of research. Mainly, sport-based programs (Hayhurst, 2011; Hellison, 2011; Spaaij, 2012) are the focus of analysis, as well as their contribution to social wellbeing and development among youngsters in the form of gender equality, academic performance, and the improvement of social, emotional and leadership skills, among others. Some papers analyse more general education programs though (Durlak et al., 2011).

Cluster #8 gathers papers that look into **Consumer Ethical Perception** (Newell and Frynas, 2007). As a matter of fact, 27.8% of the articles belonging to this cluster were published in marketing journals. In spite of the fact that the power of social initiatives may differentiate socially responsible firms (Simmons and Becker-Olsen, 2006), the motives perceived by consumers of corporate social responsibility efforts may have either positive or negative effects (Vlachos et al., 2009). Real corporate philanthropy (Sen, 2006) perceived by customers as proactive initiatives (Becker-Olsen et al., 2006) creates customer gratitude (Palmatier et al., 2009) which will lead to long-lasting customer relationships and better financial performance (Brammer and Millington, 2008). On the contrary, the perception and awareness of insincere and reactive social initiatives will be punished (Becker-Olsen et al., 2006).

Cluster #9 analyses different **Environmental Issues** (Ambec and Lanoie, 2008; Pivato et al., 2008; Adema and Roehl, 2010). Take the example of Collins et al. (2009) which deals with the environmental impacts of mega sport events.

Finally, cluster #10 is the smallest one and deals with **Corporate Citizenship**, specifying the activities that a sport organisation engages in to meet their social obligations (Walters and Chadwick, 2009).

The mean publication year of the papers in each cluster is also shown in Table 5. On the one hand, clusters #3, #5 (2016), and #2 (2014) are the clusters with a later mean publication year, which means that they are advancing at a quicker pace than the rest of them. On the other hand, clusters #4, #8 (2007), #9 (2008), #1, and #10 (2009) have an older mean. It does not seem to suggest any pattern between the size of the cluster and the mean publication for larger clusters, given that some of them are older (#1) or more recent (#2 and #3). Conversely, in our sample, the smallest clusters are among the older clusters (#8, #9 and #10).

### Intellectual Turning Points in Economic and Social Value in the Sport Industry

Co-citation clusters are common thematic research structures. Every single paper is represented in the graphic by means of a node or dot. In this regard, those that connect different clusters can be considered intellectual turning points (Chen and Song, 2019). The importance of a node or dot when connecting other nodes is measured by means of betweenness centrality which quantifies the number of times a node behaves as if it were a bridge along the shortest path between two other nodes. Therefore, a node with high levels of betweenness centrality can be considered as an essential connector between two or more nodes (Chen and Song, 2019). According to a bibliometric perspective, betweenness centrality is correlated with long-term future citations of the paper (Shibata et al., 2007).

Those nodes whose betweenness centrality is higher than 0.10 can be considered, according to social network theory, as high betweenness centrality nodes. These usually tend to find themselves on the paths that connect different clusters (Chen and Song, 2019). Table 6 shows 13 papers whose betweenness centrality is higher than 0.10 on economic and social value in the sport industry. These publications can be considered the intellectual backbone of the field. Table 6 shows the economic and social value in the sport industry network. The average number of papers with high betweenness centrality (>0.10) per cluster is 1.3 per cluster. The research areas that spread the most knowledge and have the most intellectual turning points are clusters #6 and #7 (with 5 and 4 publications respectively with betweenness centrality higher than 0.10). Clusters #1, #2, #3 and #4 have only one turning point among their notes. Finally, the least connected research areas are clusters #5, #8, #9 and #10 with no intellectual turning point.

**TABLE 6:** Intellectual turning point cited articles in economic and social value in the sport industry.

**Centrality**	**Cluster**	**Author**	**Title**	**Year**	**Source**
0.18	6	Alexandris and Tsiotsou	Segmenting soccer spectators by attachment levels: a psychographic profile based on team self-expression and involvement	2012	Eur Sport Manag Q
0.17	6	Lock, Taylor, Funk and Darcy	Exploring the development of team identification	2012	J Sport Manage
0.17	6	Pérez, García de los Salmones and Rodríguez del Bosque	The effect of corporate associations on consumer behaviour	2013	Eur J Marketing
0.16	1	Babiak and Wolfe	Determinants of corporate social responsibility in professional sport: internal and external factors	2009	J Sport Manage
0.15	2	Anagnostopoulos and Shilbury	Implementing corporate social responsibility in English football: towards multi-theoretical integration	2013	Sport Bus Manag
0.15	6	Mazodier and Rezaee	Are sponsorship announcements good news for the shareholders? Evidence from international stock exchanges	2013	J Acad Market Sci
0.15	6	Norman	Saturday night’s alright for tweeting: cultural citizenship, collective discussion, and the new media consumption/production of Hockey Day in Canada	2012	Sociol Sport J
0.15	7	Hayhurst	Corporatising sport, gender and development: postcolonial IR feminisms, transnational private governance and global corporate social engagement	2011	Third World Q
0.14	4	Breitbarth and Harris	The role of corporate social responsibility in the football business: towards the development of a conceptual model	2008	Eur Sport Manag Q
0.12	7	Durlak, Weissberg, Dymnicki, Taylor and Schellinger	The impact of enhancing students’ social and emotional learning: a meta−analysis of school−based universal interventions	2011	Child Dev
0.12	7	Spaaij	Building social and cultural capital among young people in disadvantaged communities: lessons from a Brazilian sport-based intervention program	2012	Sport Educ Soc
0.11	3	Gordon and Doyle	Teaching personal and social responsibility and transfer of learning: opportunities and challenges for teachers and coaches	2015	J Teach Phys Educ
0.11	7	Hellison	Teaching personal and social responsibility through physical activity	2011	Book

The four highest betweenness centrality (>0.15) papers are shown in Table 7. These publications act as bridges between different clusters, they are the ones that have helped the most to propagate the field of research on economic and financial value in the sport industry. These papers are connected to a great number of research articles in the field. This is to say that a large number of researchers consider these articles to be an intellectual resource.

**TABLE 7:** Top four intellectual turning point cited articles in economic and social value in the sport industry.

**Centrality**	**Cluster**	**Author**	**Title**	**Year**	**Source**	**Study type**	**Country**	**Sport**	**Methodology**
0.18	6	Alexandris and Tsiotsou	Segmenting soccer spectators by attachment levels: a psychographic profile based on team self-expression and involvement	2012	Eur Sport Manag Q	Theoretical and empirical	Greece	Football	Quantitative
0.17	6	Lock, Taylor, Funk and Darcy	Exploring the development of team identification	2012	J Sport Manage	Theoretical and empirical	Australia	Football	Qualitative
0.17	6	Pérez, García de los Salmones and Rodríguez del Bosque	The effect of corporate associations on consumer behaviour	2013	Eur J Marketing	Theoretical and empirical	Spain	*Financial services	Quantitative
0.16	1	Babiak and Wolfe,	Determinants of corporate social responsibility in professional sport: internal and external factors	2009	J Sport Manage	Theoretical and empirical	United States	NFL, MLB, NHL and NBA	Qualitative

*It does not deal with sport industry.

Out of the four papers with the highest betweenness centrality, three belong to cluster #6 (Alexandris and Tsiotsou, 2012; Lock et al., 2012; Pérez et al., 2013). As a matter of fact, they are also the three publications with the highest betweenness centrality. This makes sense given that emotional value is a key feature in sports which leads to high levels of team identification, attachment, and loyalty.

The highest publication in terms of betweenness centrality is a paper by Alexandris and Tsiotsou (2012). This article provides empirical evidence for the application of the sport attachment construct as a criterion to segment football spectators. By means of a cluster analysis and a discriminant analysis, and the use of self-expression and team involvement variables, Greek football spectators are segmented into two psychographic profiles: low and high fan attached spectators. The latter has also high scores in their self-expression and team involvement. Therefore, these individuals can be described as people who feel that their team can help them to build their self-identity, given that they are both cognitively and emotionally involved. They consider their team as part of their everyday life. This profile is more likely to be men rather than women, with a university degree and between 22 and 26 years old. The authors conclude that symbolic meaning is placed on sports, that fans should share values with their team so as to feel identified and that sport performance is not the only outcome that affects fan enjoyment.

Secondly, Lock et al. (2012) integrate both social identity theory and the Psychological Continuum Model (PCM) to explore how team identification develops in relation to Sydney Football club, a new entity created in 2004 playing the brand new A-League, the Australian football competition. Their main research items have to do with the processes that lead to developments in team identification and the manifestations of developed team identification. They conclude that the development of identification transitioned from an external to an internal state, that the internal meaning transitioned from a group of players to a set of public developed personas, that searching in the media is a team directed outcome of developed identification and that developed identification was manifested when members promote the club to other people.

Thirdly, Pérez et al. (2013) study the relationship between corporate associations and loyalty by means of the analysis of the role of satisfaction and the consumer identification in the financial services industry. They conclude that CSR contributes to building the identification with the company and satisfaction.

Finally, the fourth paper with a higher betweenness centrality value is by Babiak and Wolfe (2009), which belongs to cluster #1 and deals with the CSR theoretical framework. Belonging to this cluster and, at the same time, being an intellectual turning point makes sense, given that this cluster is part of the setting of the conceptual model of CSR in sports. In actual fact, this article identifies internal and external determinants of CSR in professional sport, with the latter being more important than the former. Particularly, key constituents, the interconnectedness of the field, and pressures from the league are shown as the more important external determinants. This qualitative research focuses on four professional teams, each one of them playing in the National Football League (NFL), Major League Baseball (MLB), National Hockey League (NHL) and National Basket Association (NBA); and located in one Midwestern American city. All in all, a framework of CSB in sports is proposed which considers both external pressures and internal resources.

### Burst Detection in Economic and Social Value in the Sport Industry

The number of publications and number of citations are relevant indicators to infer the impact of a discipline on the scientific academia but we cannot measure its influence or density, as well as its evolution over time. A deep analysis of the obtained clusters about publishing relations is necessary. Citation burst is a more relevant indicator to identify the most active research area or areas during a period of time. Running the specific burst algorithm introduced by Kleinberg (2003), CiteSpace detects changes in a variable relative to others in the same population during time periods. If the number of citations received by a publication increases considerably during a specific period of time, it can be said that a citation burst has occurred. In a brief space of time this publication has attracted great attention from other academic colleagues. For this reason, a cluster that has several nodes with strong citation bursts indicates that it is an emergent and active research area (Chen et al., 2009).

Table 8 shows the results of the burst detection analysis and identifies the 14 papers with the largest citation bursts within the economic and social value in the sport industry field between 2000 and 2020. The 14 papers were found to have citation bursts by the Kleinberg (2003) algorithm. The rest of the papers did not show enough bursts. We should note that the analysis is right censoring for burst periods in 2020, therefore, we do not know the end date of the burst periods for these publications. The area with the most burst papers is the CSR theoretical framework (cluster #1). Seven publications in this area show strong citation bursts. After these, the next cluster with four citation burst papers is #2, related to CSR case studies. Cluster #4 deals with Strategic CSR and has three burst papers. The average number of burst papers per cluster is 1.4. Nonetheless, only three clusters out of 10 have burst papers.

**TABLE 8:** Burst cited papers in the economic and social value in the sport industry field.

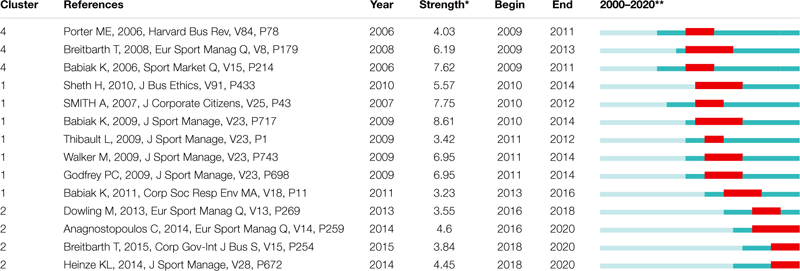

*Strength of the burst of a document (citation burst in a certain period) based on the Kleinberg (2003) algorithm. **Red line segment represents the time period of time in which a reference was found to have a burst, indicating the beginning year and the ending year of the duration of the burst.

All the burst papers are dated between 2006 and 2015, in spite of the fact that the database includes publications from 2000 to 2020. Between 2006 and 2010, the average number of burst papers per year is 1.80. From 2011 to 2015, the average is 1.0. The most prolific year with regard to burst papers is 2009 with 4. There has been an absence of burst papers since 2015. This is a paradox, given that the pace of citing publications has greatly increased since 2015 (Figure 1). The average interval between the publication of burst papers and its maximum interest is 2.21 years. One paper burst the very same year in which it was published (Sheth and Babiak, 2010) and two publications just the next year after its publication (Breitbarth and Harris, 2008; Babiak and Wolfe, 2009). These three papers along with Anagnostopoulos et al. (2014) have the longest period time (4 years) that a publication has been burst.

According to Hou et al. (2018) burst paper detection reveals research trends in a field of knowledge. In this regard, there have been three trends in the field of economic and social value in sport industry research. Table 9 shows the three trends classified by cluster and indicates the number of burst papers, the year when this trend started [Min (year)] and finished [Max (year)], the mean year, the mean strength value, the year when that trend started [Min (begin)] and the year that the trend finished [Max (end)].

**TABLE 9:** Burst cited papers per cluster in the economic and social value in the sport industry field.

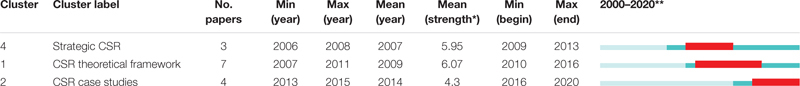

*Mean strength of the burst of a document of the cluster (citation burst in a certain period) based on the Kleinberg (2003) algorithm. **Red line segment represents the mean period of time in which a cluster was found to have a burst, indicating the minimum beginning year and the maximum ending year of the duration of the burst in a cluster.

The analysis of Table 9 shows that the first trend is compound by three papers of cluster #4, which deals with strategic CSR. The publication of burst papers started in 2006 and finished in 2008. Nonetheless, they burst from 2009 through to 2013. Just one year after the inception in the writing of the first trend, the writing of the second one started within cluster #1. This second trend comprises seven papers that were written from 2007 to 2011 and develops the theoretical framework of CSR in sports. These papers burst from 2010 to 2016. Just when the second trend vanished, in 2016, the third trend burst within cluster #2. This last trend deals with CSR case studies that were written from 2013 (2 years after the end of the writing of the second trend) through to 2015.

All things considered, the first two trends, that overlap partly in time, show that academics initially found great interest in achieving eminently theoretical understanding, namely, setting the conceptual framework and the foundation of CSR in the sport industry as a strategic feature. Once theory and strategy were well-established, researchers showed great interest in the implementation of CSR by means of the analysis of sport entities cases, which compounded the third and last trend. For the time being, this third trend is still alive given that three out of the four papers that burst, are still so in 2020.

## Discussion and Conclusion

In this article, we have carried out a co-citation bibliometric analysis with the aim of mapping the intellectual structure of the economic and social value in the sport industry field. Our findings include quantitative rigour and contribute to further development of the field in the following ways.

Firstly, we have delineated the principal research areas within the field of economic and social value in sport industry research: CSR theoretical framework; CSR case studies; TPSR; strategic CSR; sponsorship; emotional value; educative programs; consumer ethical perception; environmental issue; and corporate citizenship.

In comparison with Maestre-Matos et al. (2020) who perform a bibliometric analysis about shared value (defined as the integration of the generation of economic and social value) but without segmenting by sport industry, some more general principal research areas are some how shared, namely CSR theoretical framework, CSR case studies, strategic CSR, and environmental issue. Nonetheless, given that our research focuses on the sport industry, specific research areas have been pinpointed: TPSR; sponsorship; emotional value; educative programs; consumer ethical perception; and corporate citizenship. This makes sense, given that, for instance, sponsorship is a key feature of the sport industry and many papers deal with this topic. On the other hand, emotional value is also a relevant idiosyncrasy of sport which is related to the emotional attachment that supporters have to a sport institution.

Secondly, the papers that form the intellectual backbone of economic and social value in sport industry research have been identified. Mapping these papers reveals the paths and connections through which economic and social value in the sport industry has disseminated. The turning points represent the bases of the knowledge of the field of research. Their identification allows academia to obtain a quicker understanding of the field, from where to begin the research process. For instance, emotional value researchers who want to know about brand identity, team identification and attachment as well as economic and social value in the sport industry, should begin their literature review by means of reading Alexandris and Tsiotsou (2012) and Lock et al. (2012). Therefore, these papers are the main source of knowledge in this specific field. As a consequence, they should also be the starting point on which to base the knowledge of future research in this area. In a similar way, future research on CSR in the sports industry should establish its knowledge based on the Babiak and Wolfe (2009). This way of proceeding is a remarkable time saving strategy for researchers, primarily during the early stages of developing the conceptual framework of research given that the engines of knowledge of the field are taken as references.

The fact that one of the four papers that are considered intellectual turning point articles in economic and social value in the sport industry (Pérez et al., 2013) does not deal with the sport industry, confirms the main conclusion of this article: the existence of a literature gap in regard to quantitative methodology to assess social value in the sport industry. As a matter of fact, Pérez et al. (2013) is a quantitative analysis. Due to this lack of quantitative methodological literature in the sport industry, authors that research this issue feel forced to consult other sources of knowledge to be nourished with quantitative studies and not only qualitative (which abound in regard to social value in the sport industry). Therefore, scholars that research this field have applied quantitative methodologies used in other industries, due to the lack of a standardised methodology in the sport industry so as to assess social value.

Thirdly, burst papers, in other words, articles that have attracted extraordinary attention from the scientific community during a discrete time period, have been identified. Detecting burst papers provides an image of the changing situation of the literature on economic and social value in the sport industry. This image is essential because it provides the guidance needed so as to allow academics to, on the one hand, design new research that advances the identified trends and, on the other hand, open new research fields based on these trends.

Two trends overlapped from 2009 to 2016 which set the conceptual framework of CSR and analyse CSR as a strategy. The writing process of these two initial trends took place between 2006 and 2011. Once the theory and the strategic feature of CSR were well-established, researchers showed great interest in the implementation of CSR by means of the analysis of sport entities cases. Therefore, a new trend, the third and last one, emerged: papers written in 2013, 2014, and 2015, dealing with CSR case studies, burst from 2016 onwards. For the time being, this third trend is still bursting or alive given that three out of the four papers that burst, are still so in 2020.

This means that there has been no new burst paper over the last 5 years (since 2015), in spite of the fact that the mean time from publication to maximum interest for a burst paper is 2.21 years. This is a paradox because the number of citing papers on economic and social value in the sport industry field (Figure 1) written from 2016 to 2020 is higher (276 citing papers) than those written from 2000 to 2015 (218 documents). How is that possible? The methodology used in burst papers provides insight in this regard.

Out of the 14 burst papers, only three use qualitative analysis; but never alone, always accompanied by qualitative methodologies (Walker and Kent, 2009; Sheth and Babiak, 2010; Babiak and Trendafilova, 2011). All these papers belong to cluster #1, that deals with the theoretical framework, and the period that these remained burst is longer than the mean period of the 14 papers. The rest of the burst papers use either theoretical or qualitative approaches, which have indeed limitations. In actual fact, CSR case studies are the thematic topic of cluster (#4), with some papers that are still bursting.

The analysis of these trends suggests future lines of research which should cover the gap in regard to the measurement of social value in sports. This would allow the increase of quantitative research in the field of economic and social value in the sport industry. This has been the case with some other industries, where over the last few years some quantitative indicators have been established so as to assess social value, integrating it along with economic performance indicators. Take the example of the banking industry, where literature has been written in this regard, proposing quantitative models and developing standardised measurements to integrate both the economic and social value of financial institutions (San-José et al., 2012, 2014; Torres-Pruñonosa et al., 2012). The existence of this quantitative theoretical framework has allowed researchers to quantitatively analyse the social efficiency of various financial intermediaries (Gutiérrez-Goiria et al., 2017; Bachiller and García-Lacalle, 2018; San-José et al., 2018, 2020).

Therefore, given that financial and economic indicators are easily recognisable when reviewing the existing literature, the development of measurements for social value of sport entities is a research opportunity that may be turned into the next burst paper in this field of knowledge. This quantitative methodology will be cited by many scholars that are researching this increasing field of knowledge. To all intents and purposes, this new methodological approach would consist of adapting either the monetisation of social value or social accounting models to the sports framework (Retolaza et al., 2016).

In actual fact, if these quantitative indicators were established, similar quantitative and not only qualitative research (Mendizábal et al., 2020) that has been done within stakeholders theory (Freeman, 1984; Freeman et al., 2010) could be developed in the sport industry. In this regard, the multi-fiduciary theory of stakeholders (Goodpaster, 1991; Boatright, 2008) establishes the relationship between various stakeholders (that are not only shareholders) that are the principals and the agents (those who have fiduciary responsibility behind the group of stakeholders). Therefore, the agent will be legitimately obligated to respond to the stakeholders’ interest. Nonetheless, Jensen (2002) argues that it is impossible to manage the interest of all stakeholders given that there is not a person with enough legitimacy to monitor the decision-making agent, because those that are the controllers (several stakeholders with autonomy) have dispersed and incompatible interests. This is what is commonly called among scholars Jensen’s ‘governance problem’.

Savings banks can be considered both a prototypic case of the multi-stakeholders governance^2^ and a paradigmatic case of Jensen’s ‘governance problem’. According to this approach, they were supposed to collapse within a very short period of time. Nonetheless, they have survived over the last two centuries. According to this paradigm, organisation with a large diversity of interests and complexity in their control, such as savings banks, were expected to be significantly less efficient than commercial banks, given than the later have a shareholder based model. As a matter of fact, San-José et al. (2014) analyse the economic, social, and overall efficiency of savings banks against commercial banks. By means of a quantitative and integrated model, results refute Jensen’s ‘problem of governance’, given that there is not significantly less efficiency in savings banks in comparison to commercial banks during both the housing boom (Raya et al., 2017) and recession years. Savings banks seem to be a case of auto-regulation in the governance of the common pool resources (Ostrom et al., 1999; Ostrom, 2015).

Similarly, Spanish football clubs (namely, Futbol Club Barcelona, Real Madrid Club de Fútbol, Athletic Club and Club Atlético Osasuna) are also prototypic cases of the non-shareholders governance and could be paradigmatic cases of Jensen’s ‘governance problem’. Are they less efficient than shareholder governed football companies? To this aim, it is necessary to cover the lack of standardised indicators in order to analyse the overall (including not only economic but also social) efficiency of football clubs against Sport Stock Corporations [what is called ‘Sociedades Anónimas Deportivas’ (SAD) according to the Spanish legislation]. As a matter of fact, this analysis would be very noteworthy given that non-shareholder governed clubs encapsulate the two most laureate teams in Spain (FC Barcelona and Real Madrid CF), a historic team (Athletic Club, the only football team along with FC Barcelona and Real Madrid CF that has never been relegated to the Spanish second division) and a club who has had several relegations and promotions over the last few years (CA Osasuna). Therefore, these entities have different sizes and profiles and, consequently, they could be a good sample to see if Jensen’s ‘problem of governance’ is again refuted (such as it happened in the case of savings banks) in comparison with Spanish Sport Stock Corporations. In actual fact, given that FC Barcelona and Real Madrid CF usually play in the UEFA Champions League and Athletic Club usually play in European football competitions, the comparison with European shareholder governed football companies could also be carried out.

Fourthly, we have also examined the scientific journals that have contributed the most to this field of knowledge. Actually, the 494 citing papers were published in 201 different academic journals. The journals that have published 10 citing papers or more (Table 1) in regard to the field of economic and social value in the sport industry are: *European Sport Management Quarterly* (39), *Journal of Sport Management* (34), *Sport Management Review* (29), *Journal of Teaching in Physical Education* (18), *International Journal of Sports Marketing Sponsorship* (17), *Sport in Society* (16), *Journal of Business Ethics* (11) and *Sustainability* (10). Out of these eight journals, one is currently included in the first quartile according to the Impact Factor of Journal Citation Reports, four in the second quartile, two in the third quartile, and one in the fourth quartile. The results obtained in regard to centrality (Table 6), allow us to identify which journals have published the most relevant or influential articles in this field, given that they are considered intellectual turning points: *European Sport Management Quarterly* (2), *Journal of Sport Management* (2), *European Journal of Marketing* (1), *Sport Business Management* (1), *Journal of the Academy of Marketing Science* (1), *Sociology of Sport Journal* (1), *Third World Quarterly* (1), *Child Development* (1), *Sport Education and Society* (1), and *Journal of Teaching in Physical Education* (1). In the regards to burst papers (Table 8), the journals that have published some of them are: *Journal of Sport Management* (5), *European Sport Management Quarterly* (3), *Harvard Business Review* (1), *Sport Marketing Quarterly* (1), *Journal of Business Ethics* (1), *Journal of Corporate Citizenship* (1), *Corporate Social Responsibility and Environmental Management* (1) and *Corporate Governance International Journal of Business in Society* (1). Therefore, there is evidence that the papers that contain more intellectual turning points and burst papers are two specialised sports journals: *European Sport Management Quarterly* and *Journal of Sport Management.* Knowing where the most influential research papers in economic and social value in the sport industry have been published will be useful for future researchers, given that it will save time when conducting the literature review.

Fifthly, bibliometric analysis has the advantage of giving order to an immense amount of data; a meaningful order, in fact, from which good quantitative and approximate qualitative evaluations can be extracted. Nevertheless, it also has some limitations that have been recurring for decades (Smith, 1981). A high level of involvement and, therefore, subjective participation of those who carry out the analysis, is necessary to interpret the results obtained. Thus, bibliometric techniques must be accompanied by the intellectual refinement that requires, for example, an extensive bibliographic review on the subject, its synthesis, and/or discussion among scholars (van Raan, 2004; Bergstrom et al., 2008; Zupic and Èater, 2015). In order to achieve a consistent set of indicators that allows researchers to reach a reasonable conclusion, the researcher needs to make some technical decisions in the parameters of the chosen database and the analytical software (e.g., restrictions of language, journals, time period, normalisation of cluster labelling, verification of final data to debug errors, etc.) (van Raan, 2004). There is also the problem that older publications will have more presence than newer ones simply because of the delay in which citations are entered (Vogel and Güttel, 2013) or the consideration or not of self-citations (Glänzel et al., 2006; Peroni et al., 2020).

### Limitations

Finally, we recognise that the use of a single database (SSCI) limits the research. It is impossible to cover the entire field of knowledge of the subject that is going to be analysed without taking into consideration other recognised databases (Scopus, Dimensions, etc.) (Meho and Yang, 2007); or, without the data offered by techniques applied to the analysis of scientific publications and activity on the internet such as Webometrics or Altmetrics (Thelwall, 2009, 2018; Brigham, 2014; Roemer and Borchardt, 2015). Nevertheless, in this case, the value that SSCI has in social sciences bibliometric analysis is well recognised and, therefore, the results offered are well supported. Logically these limitations must be taken into account when considering the results obtained in this research.

## Data Availability Statement

The original contributions presented in the study are included in the article/Supplementary Material, further inquiries can be directed to the corresponding author/s.

## Author Contributions

All authors listed have made a substantial, direct and intellectual contribution to the work, and approved it for publication.

## Conflict of Interest

The authors declare that the research was conducted in the absence of any commercial or financial relationships that could be construed as a potential conflict of interest.

## Footnotes

1 We focus on the social sciences view of sports, not on medical, psychological, or engineering approaches which are included in the Science Citation Index (SCI) database.

2 Savings banks are based on a multi-fiduciary model that takes into account work, human resources, or society decision legitimacy, among others, as items to determine the governance of the institution (Boatright, 2008; García-Cestona and Surroca, 2008; Retolaza et al., 2018). Conversely, commercial banks are based on the property right model which establishes capital as the key to governance.
