# Supplementary material for: The Sources of Knowledge of the Economic and Social Value in Sport Industry Research: A Co-citation Analysis
Source: Front Psychol. 2020 Dec 29;11:629951. doi: 10.3389/fpsyg.2020.629951 (PMC7802761; doi:10.3389/fpsyg.2020.629951)
Supplement: Supplementary file 2 [file Table_2.DOCX]

Supplementary Material

**Supplementary Figure 1.** Network Summary.

| **Cluster** | **Burst** | **Centrality** | **Author** | **Year** | **Source** | **Vol** | **Page** |
| --- | --- | --- | --- | --- | --- | --- | --- |
| 1 | 8.61 | 0.16 | Babiak K | 2009 | J SPORT MANAGE | 23 | 717 |
| 1 | 5.57 | 0.09 | Sheth H | 2010 | J BUS ETHICS | 91 | 433 |
| 1 | 6.95 | 0.03 | Walker M | 2009 | J SPORT MANAGE | 23 | 743 |
| 1 | 3.23 | 0.03 | Babiak K | 2011 | CORP SOC RESP ENV MA | 18 | 11 |
| 1 | 7.75 | 0.01 | SMITH A | 2007 | J CORPORATE CITIZENS | 25 | 43 |
| 1 | 6.95 | 0.01 | Godfrey PC | 2009 | J SPORT MANAGE | 23 | 698 |
| 1 | 3.42 | 0.01 | Thibault L | 2009 | J SPORT MANAGE | 23 | 1 |
| 1 |  | 0.01 | Kaufman P | 2010 | J SPORT SOC ISSUES | 34 | 154 |
| 1 |  | 0.01 | Aguinis H | 2012 | J MANAGE | 38 | 932 |
| 1 |  | 0.01 | Hums MA | 2010 | J SPORT MANAGE | 24 | 1 |
| 1 |  | 0.01 | Jamali D | 2009 | J BUS ETHICS | 84 | 277 |
| 1 |  | 0.01 | Breitbarth T | 2011 | THUNDERBIRD INT BUS | 53 | 721 |
| 1 |  | 0.00 | Levermore R | 2010 | THIRD WORLD Q | 31 | 223 |
| 1 |  | 0.00 | Bradish C | 2009 | J SPORT MANAGE | 23 | 691 |
| 1 |  | 0.00 | Madrigal R | 2008 | HDB CONSUMER PSYCHOL | 0 | 857 |
| 1 |  | 0.00 | Misener L | 2009 | J SPORT MANAGE | 23 | 770 |
| 1 |  | 0.00 | Babiak KM | 2009 | EVAL PROGRAM PLANN | 32 | 1 |
| 1 |  | 0.00 | Husted BW | 2010 | BUS SOC | 49 | 201 |
| 1 |  | 0.00 | Walters G | 2010 | J MANAGE ORGAN | 16 | 566 |
| 1 |  | 0.00 | Du SL | 2008 | J CONSUM RES | 35 | 483 |
| 1 |  | 0.00 | Siegel DS | 2009 | ACAD MANAGE PERSPECT | 23 | 5 |
| 1 |  | 0.00 | Bhattacharya CB | 2009 | J BUS ETHICS | 85 | 257 |
| 1 |  | 0.00 | Alexandar L | 2011 | SPORTS TEAMS SOCIAL | 0 | 0 |
| 1 |  | 0.00 | Walker M | 2010 | J BUS ETHICS | 95 | 659 |
| 1 |  | 0.00 | Andrews R | 2010 | J PUBL ADM RES THEOR | 20 | 679 |
| 1 |  | 0.00 | Ratten V | 2010 | J MANAGE ORGAN | 16 | 488 |
| 1 |  | 0.00 | Gaffney C | 2010 | J LAT AM GEOGR | 9 | 7 |
| 1 |  | 0.00 | Soderlund M | 2006 | INT J SERV IND MANAG | 17 | 76 |
| 1 |  | 0.00 | Heere B | 2011 | J SPORT MANAGE | 25 | 606 |
| 1 |  | 0.00 | Diehl D | 2007 | RW JOHNSON ANTHOLOGY | 11 | 1 |
| 1 |  | 0.00 | Ponte S | 2009 | THIRD WORLD Q | 30 | 301 |
| 1 |  | 0.00 | KIM Y-J | 2007 | INT J SPORT MARK SPO | 8 | 310 |
| 1 |  | 0.00 | Irwin CC | 2010 | J SCHOOL HEALTH | 80 | 333 |
| 1 |  | 0.00 | RATTEN V | 2010 | INT ENTREPRENEURSHIP | 0 | 0 |
| 1 |  | 0.00 | Hoglund K | 2008 | THIRD WORLD Q | 29 | 805 |
| 1 |  | 0.00 | Loakimidis M | 2007 | SPORT J | 10 | 0 |
| 1 |  | 0.00 | Cornelissen S | 2008 | SPORT SOC | 11 | 481 |
| 1 |  | 0.00 | Rupp DE | 2013 | PERS PSYCHOL | 66 | 895 |
| 1 |  | 0.00 | Walker M | 2011 | SPORT MANAG REV | 14 | 153 |
| 1 |  | 0.00 | Seitanidi MM | 2009 | J BUS ETHICS | 85 | 413 |
| 1 |  | 0.00 | *DIV AD COMM HLTH | 2010 | ALC PUBL HLTH FACT S | 0 | 0 |
| 1 |  | 0.00 | *GLOB FUND | 2010 | GLOB FUND 2010 INN I | 0 | 0 |
| 1 |  | 0.00 | *IND COLTS | 2010 | LUC OIL STAD POL | 0 | 0 |
| 1 |  | 0.00 | Andrews DL | 2012 | SPORTING | 0 | 1 |
| 1 |  | 0.00 | Ariely D | 2009 | AM ECON REV | 99 | 544 |
| 1 |  | 0.00 | Anderson E D | 2009 | SPORT MANAGEMENT REV | 12 | 3 |
| 1 |  | 0.00 | **BritishOlympicAssociation | 2011 | FTSE BOA IN | 0 | 0 |
| 1 |  | 0.00 | **InternationalOlympicCommittee | 2009 | REPORT OF THE 2016 I | 0 | 0 |
| 1 |  | 0.00 | **NationalBrandIndex | 2009 | EV NAT BRAND COUNTR | 0 | 0 |
| 1 |  | 0.00 | Anagnostopoulos C | 2011 | Soccer and Society | 12 | 249 |
| 1 |  | 0.00 | Andreff W | 2010 | EC INT SPORT | 0 | 0 |
| 1 |  | 0.00 | **InternationalOlympicCommittee | 2011 | SPONS | 0 | 0 |
| 1 |  | 0.00 | Alegi P | 2008 | AFR STUD-UK | 67 | 397 |
| 1 |  | 0.00 | *AM COLL HLTH ASS | 2010 | REF GROUP EX SUMM | 0 | 0 |
| 1 |  | 0.00 | **UN Educational Scientific… | 2011 | SPORT PEAC DEV | 0 | 0 |
| 1 |  | 0.00 | Andriessen K | 2009 | CRISIS | 30 | 144 |
| 1 |  | 0.00 | **NationalFootballLeague | 2010 | SUPER BOWL COMMUNITY | 0 | 0 |
| 1 |  | 0.00 | AEG | 2010 | AEGS 2010 ENV SUST R | 0 | 0 |
| 1 |  | 0.00 | **CharityCommissionforEnglandandWales | 2012 | FDN MOD CONST CHAR I | 0 | 0 |
| 1 |  | 0.00 | Anagnostopoulos C | 2013 | HANDBOOK RESEARCH SPORT BUS | 0 | 418 |
| 1 |  | 0.00 | Anagnostopoulos C | 2013 | HDB SPORT CORPORATE | 0 | 91 |
| 1 |  | 0.00 | Aldama L R P | 2009 | CORPORATE GOVERNANCE | 9 | 506 |
| 1 |  | 0.00 | **VancouverOrganizingCommittee | 2010 | 12 WAYS VANOC BUILT | 0 | 0 |
| 1 |  | 0.00 | *AR CARD | 2010 | 2010 A TO Z GUID | 0 | 0 |
| 1 |  | 0.00 | *SEATTL SEAH | 2010 | ALC POL | 0 | 0 |
| 1 |  | 0.00 | **UN Sport for Develop … | 2008 | SPORT DEV PEAC PRACT | 0 | 0 |
| 1 |  | 0.00 | **NationalBasketballAssociation | 2010 | NBA GREEN WEEK 2010 | 0 | 0 |
| 1 |  | 0.00 | **NationalHockeyLeague | 2010 | NHL ANNOUNCES GREEN | 0 | 0 |
| 1 |  | 0.00 | Auerswald P | 2009 | STANFORD SOCIAL INNO | 0 | 51 |
| 2 |  | 0.15 | Anagnostopoulos C | 2013 | SPORT BUS MANAG | 3 | 268 |
| 2 | 4.45 | 0.05 | Heinze KL | 2014 | J SPORT MANAGE | 28 | 672 |
| 2 |  | 0.05 | Kolyperas D | 2016 | J SPORT MANAGE | 30 | 702 |
| 2 | 4.60 | 0.03 | Anagnostopoulos C | 2014 | EUR SPORT MANAG Q | 14 | 259 |
| 2 |  | 0.03 | Kolyperas D | 2015 | CORP GOV-INT J BUS S | 15 | 177 |
| 2 | 3.84 | 0.02 | Breitbarth T | 2015 | CORP GOV-INT J BUS S | 15 | 254 |
| 2 | 3.55 | 0.02 | Dowling M | 2013 | EUR SPORT MANAG Q | 13 | 269 |
| 2 |  | 0.02 | Kihl L | 2014 | J SPORT MANAGE | 28 | 324 |
| 2 |  | 0.02 | Lacey R | 2016 | SPORT MARKET Q | 25 | 21 |
| 2 |  | 0.02 | Schyvinck C | 2018 | SPORT MANAG REV | 21 | 347 |
| 2 |  | 0.01 | Babiak K | 2012 | J SPORT MANAGE | 26 | 159 |
| 2 |  | 0.01 | Filo K | 2015 | SPORT MANAG REV | 18 | 166 |
| 2 |  | 0.01 | Bingham T | 2013 | VOLUNTAS | 24 | 606 |
| 2 |  | 0.01 | Reiche D | 2014 | Soccer and Society | 15 | 472 |
| 2 |  | 0.01 | Schulenkorf N | 2016 | J SPORT MANAGE | 30 | 22 |
| 2 |  | 0.01 | Schulenkorf N | 2012 | SPORT MANAG REV | 15 | 1 |
| 2 |  | 0.01 | **CelticFCFoundation | 2015 | CELT FC FDN ENS REF | 0 | 0 |
| 2 |  | 0.00 | Walzel S | 2018 | J SPORT MANAGE | 32 | 511 |
| 2 |  | 0.00 | Rohde M | 2017 | EUR SPORT MANAG Q | 17 | 265 |
| 2 |  | 0.00 | Trendafilova S | 2013 | SPORT MANAG REV | 16 | 298 |
| 2 |  | 0.00 | Acero I | 2017 | CORP GOV-INT J BUS S | 17 | 511 |
| 2 |  | 0.00 | Inoue Y | 2011 | J SPORT MANAGE | 25 | 531 |
| 2 |  | 0.00 | Abeza G | 2015 | J SPORT MANAGE | 29 | 601 |
| 2 |  | 0.00 | Yin R K | 2014 | CASE STUDY RES DESIG | 0 | 0 |
| 2 |  | 0.00 | Dimitropoulos P | 2016 | EUR SPORT MANAG Q | 16 | 459 |
| 2 |  | 0.00 | Hayhurst LMC | 2016 | J SPORT SOC ISSUES | 40 | 522 |
| 2 |  | 0.00 | Sparvero E | 2014 | J APPL SPORT MANAG | 6 | 98 |
| 2 |  | 0.00 | Walker M | 2017 | J BUS ETHICS | 143 | 53 |
| 2 |  | 0.00 | Trendafilova S | 2013 | INT J SPORT MANAGEME | 13 | 1 |
| 2 |  | 0.00 | Hills S | 2019 | SPORT MANAG REV | 22 | 126 |
| 2 |  | 0.00 | Inoue Yuhei | 2015 | INT REV PUBLIC NONPR | 12 | 189 |
| 2 |  | 0.00 | Breitbarth T | 2019 | EUR SPORT MANAG Q | 19 | 1 |
| 2 |  | 0.00 | Benson P | 2017 | J SPORT SOC ISSUES | 41 | 307 |
| 2 |  | 0.00 | Humphreys A | 2018 | J CONSUM RES | 44 | 1274 |
| 2 |  | 0.00 | Sotiriadou P | 2017 | J SPORT MANAGE | 31 | 61 |
| 2 |  | 0.00 | Darnell S C | 2015 | INT J SPORT MANAG AND MK | 16 | 5 |
| 2 |  | 0.00 | Fifka MS | 2020 | SOCCER SOC | 21 | 61 |
| 2 |  | 0.00 | Inoue Y | 2014 | J BUS ETHICS | 121 | 621 |
| 2 |  | 0.00 | Jordan JS | 2011 | J SPORT MANAGE | 25 | 229 |
| 2 |  | 0.00 | Walters G | 2014 | Soccer and Society | 15 | 828 |
| 2 |  | 0.00 | Geeraert A | 2018 | NATL SPORTS GOVERNAN | 0 | 0 |
| 2 |  | 0.00 | Burton LJ | 2015 | SPORT MANAG REV | 18 | 155 |
| 2 |  | 0.00 | Berg BK | 2015 | SPORT MANAG REV | 18 | 20 |
| 2 |  | 0.00 | Bevere L | 2014 | NATURAL CATASTROPHES | 0 | 0 |
| 2 |  | 0.00 | Abeza G | 2013 | INT J SPORT COMUN | 6 | 120 |
| 2 |  | 0.00 | Beggan DM | 2011 | DISASTER PREV MANAG | 20 | 413 |
| 2 |  | 0.00 | Abeza G | 2014 | J APPL SPORT MANAG | 6 | 103 |
| 2 |  | 0.00 | **InternationalBusinessTimes(IBT) | 2013 | IPL 2013 1 WEEK MATC | 0 | 0 |
| 2 |  | 0.00 | Agyemang K | 2013 | SPECTRUM-J BLACK MEN | 2 | 47 |
| 2 |  | 0.00 | **EuropeanAssociationforSportManagement | 2016 | 24 EUR ASS SPORT MAN | 0 | 0 |
| 2 |  | 0.00 | Bell J | 2011 | NY TIMES | 0 | 0 |
| 2 |  | 0.00 | AL-Tabbaa O | 2014 | VOLUNTAS | 25 | 657 |
| 2 |  | 0.00 | Anderson L | 2011 | SPORTS ILLUSTRATED | 0 | 0 |
| 2 |  | 0.00 | Albert N | 2013 | J CONSUM MARK | 30 | 258 |
| 3 |  | 0.11 | Gordon B | 2015 | J TEACH PHYS EDUC | 34 | 152 |
| 3 |  | 0.01 | Pozo P | 2018 | EUR PHYS EDUC REV | 24 | 56 |
| 3 |  | 0.01 | Gordon B | 2016 | J TEACH PHYS EDUC | 35 | 358 |
| 3 |  | 0.01 | Martinek T | 2016 | J PHYS EDUC RECREAT | 87 | 9 |
| 3 |  | 0.01 | Menendez-Santurio JI | 2016 | REV PSICODIDACT | 21 | 245 |
| 3 |  | 0.01 | Fernandez-Rio J | 2017 | J TEACH PHYS EDUC | 36 | 185 |
| 3 |  | 0.01 | Allen G | 2015 | QUAL RES SPORT EXERC | 7 | 53 |
| 3 |  | 0.01 | Balague G | 2016 | J PHYS EDUC RECREAT | 87 | 14 |
| 3 |  | 0.01 | Fernandez-Rio J | 2014 | J PHYS EDUC RECREAT | 85 | 3 |
| 3 |  | 0.00 | Hemphill MA | 2015 | SPORT EDUC SOC | 20 | 398 |
| 3 |  | 0.00 | Lee O | 2015 | J TEACH PHYS EDUC | 34 | 603 |
| 3 |  | 0.00 | Escarti A | 2015 | J PSYCHOL | 3 | 55 |
| 3 |  | 0.00 | Holt NL | 2017 | INT REV SPORT EXER P | 10 | 1 |
| 3 |  | 0.00 | Hastie PA | 2014 | J TEACH PHYS EDUC | 33 | 422 |
| 3 |  | 0.00 | Escarti A | 2018 | J TEACH PHYS EDUC | 37 | 12 |
| 3 |  | 0.00 | Richards KAR | 2019 | J PHYS EDUC RECREAT | 90 | 35 |
| 3 |  | 0.00 | Walsh DS | 2016 | J PHYS EDUC RECREAT | 87 | 35 |
| 3 |  | 0.00 | Bean C | 2016 | J APPL SPORT PSYCHOL | 28 | 274 |
| 3 |  | 0.00 | Richards KAR | 2020 | J TEACH PHYS EDUC | 39 | 300 |
| 3 |  | 0.00 | Gray S | 2019 | J TEACH PHYS EDUC | 38 | 347 |
| 3 |  | 0.00 | Ivy VN | 2018 | J YOUTH DEV | 13 | 162 |
| 3 |  | 0.00 | Wright PM | 2019 | J TEACH PHYS EDUC | 38 | 316 |
| 3 |  | 0.00 | Wright PM | 2020 | J TEACH PHYS EDUC | 39 | 311 |
| 3 |  | 0.00 | Jacobs JM | 2020 | J TEACH PHYS EDUC | 39 | 289 |
| 3 |  | 0.00 | Holt N L | 2016 | POSITIVE YOUTH DEV S | 0 | 0 |
| 3 |  | 0.00 | Turnnidge J | 2014 | QUEST | 66 | 203 |
| 3 |  | 0.00 | Sparkes AC | 2014 | QUALITATIVE RES METH | 0 | 0 |
| 3 |  | 0.00 | Sanchez-Alcaraz B J | 2014 | AM J SPORT SCI MED | 2 | 13 |
| 3 |  | 0.00 | Richards KAR | 2018 | J TEACH PHYS EDUC | 37 | 225 |
| 3 |  | 0.00 | Dunn RJ | 2020 | J TEACHING PHYS ED | 39 | 0 |
| 3 |  | 0.00 | Hagger MS | 2016 | REV EDUC RES | 86 | 360 |
| 3 |  | 0.00 | Patton MQ | 2015 | QUALITATIVE RES EVAL | 0 | 0 |
| 3 |  | 0.00 | Jacobs JM | 2016 | J PHYS EDUC RECREAT | 87 | 18 |
| 3 |  | 0.00 | Cryan M | 2017 | PHYS EDUC-US | 74 | 127 |
| 3 |  | 0.00 | Hemphill MA | 2016 | J TEACH PHYS EDUC | 35 | 263 |
| 3 |  | 0.00 | Jacobs JM | 2018 | QUEST | 70 | 81 |
| 3 |  | 0.00 | Casey A | 2018 | PHYS EDUC SPORT PEDA | 23 | 294 |
| 3 |  | 0.00 | Severinsen G | 2014 | ASIA PACIFIC J HLTH | 5 | 83 |
| 3 |  | 0.00 | Casey A | 2014 | PHYS EDUC SPORT PEDA | 19 | 18 |
| 3 |  | 0.00 | Barnes TN | 2014 | AGGRESS VIOLENT BEH | 19 | 311 |
| 3 |  | 0.00 | Baptista C | 2019 | INT J SPORTS SCI | 9 | 8 |
| 3 |  | 0.00 | Metzler M | 2017 | INSTRUCTIONAL MODELS | 0 | 0 |
| 3 |  | 0.00 | Beale A | 2016 | J PHYS EDUC RECREAT | 87 | 31 |
| 3 |  | 0.00 | Landi D | 2016 | J TEACH PHYS EDUC | 35 | 400 |
| 3 |  | 0.00 | Agans JP | 2016 | POSITIVE YOUTH DEV THR SPORT | 0 | 34 |
| 4 | 6.19 | 0.14 | Breitbarth T | 2008 | EUR SPORT MANAG Q | 8 | 179 |
| 4 | 4.03 | 0.03 | Porter ME | 2006 | HARVARD BUS REV | 84 | 78 |
| 4 | 7.62 | 0.01 | Babiak K | 2006 | SPORT MARKET Q | 15 | 214 |
| 4 |  | 0.01 | Godfrey PC | 2005 | ACAD MANAGE REV | 30 | 777 |
| 4 |  | 0.00 | Smith AP | 2007 | NURS ECON | 25 | 43 |
| 4 |  | 0.00 | Matten D | 2008 | ACAD MANAGE REV | 33 | 404 |
| 4 |  | 0.00 | Walker M | 2007 | BUSINESS RES YB GLOB | 14 | 926 |
| 4 |  | 0.00 | Bruch H | 2005 | MIT SLOAN MANAGE REV | 47 | 49 |
| 4 |  | 0.00 | Luo XM | 2006 | J MARKETING | 70 | 1 |
| 4 |  | 0.00 | Bhattacharya CB | 2008 | MIT SLOAN MANAGE REV | 49 | 37 |
| 4 |  | 0.00 | Montiel I | 2008 | ORGAN ENVIRON | 21 | 245 |
| 4 |  | 0.00 | BABIAK K | 2007 | M N AM SOC SPORT MAN | 0 | 0 |
| 4 |  | 0.00 | Marquis C | 2007 | ACAD MANAGE REV | 32 | 925 |
| 4 |  | 0.00 | Husted BW | 2006 | J MANAGE STUD | 43 | 75 |
| 4 |  | 0.00 | Campbell JL | 2007 | ACAD MANAGE REV | 32 | 946 |
| 4 |  | 0.00 | Vidaver-Cohen D | 2008 | BUS SOC REV | 113 | 441 |
| 4 |  | 0.00 | Collins A | 2007 | URBAN STUD | 44 | 457 |
| 4 |  | 0.00 | Godfrey PC | 2007 | J BUS ETHICS | 70 | 87 |
| 4 |  | 0.00 | Baughn C Christopher | 2007 | CSR AND ENV MANAG | 14 | 38 |
| 4 |  | 0.00 | Gan A | 2006 | J BUS ETHICS | 69 | 217 |
| 4 |  | 0.00 | *INT OL COMM | 2007 | BIRTH OL MOV | 0 | 0 |
| 4 |  | 0.00 | *NIK | 2006 | STRAT DIR LETT NIK B | 0 | 0 |
| 4 |  | 0.00 | *IOC | 2006 | IOC GUID SPORT ENV S | 0 | 0 |
| 4 |  | 0.00 | *MAJ LEAG BAS | 2008 | MLB COMM ANN REP | 0 | 0 |
| 4 |  | 0.00 | *FIFA | 2004 | ACT REP APR 2002 MAR | 0 | 0 |
| 4 |  | 0.00 | *FIM | 2008 | ENV COD | 0 | 0 |
| 4 |  | 0.00 | *AD GROUP | 2008 | SUST | 0 | 0 |
| 4 |  | 0.00 | *FIFA | 2005 | FIFA WORLD REP FOOTB | 0 | 0 |
| 4 |  | 0.00 | **DavidSuzukiFoundation | 2007 | NHL PLAYERS PUT GLOB | 0 | 0 |
| 4 |  | 0.00 | *RIGHT PLAY | 2009 | HIST RIGHT PLAY | 0 | 0 |
| 4 |  | 0.00 | *NAT BASK ASS | 2009 | LEAG LAUNCH NBA CAR | 0 | 0 |
| 4 |  | 0.00 | *MAJ LEAG BAS TEAM | 2008 | MLB GOES GREEN COLL | 0 | 0 |
| 4 |  | 0.00 | *SPORTS PHIL PROJ | 2009 | SPP MISS | 0 | 0 |
| 4 |  | 0.00 | **NationalSportingGoodsAssociation | 2006 | YOUTH PART SEL SPORT | 0 | 0 |
| 4 |  | 0.00 | *NAT HOCK LEAG GRE | 2008 | NHL AIMS PUT GLOB WA | 0 | 0 |
| 4 |  | 0.00 | *NBA | 2009 | MUT SET OP HOSP CONG | 0 | 0 |
| 4 |  | 0.00 | ** IOCFactsheet | 2008 | ENV SUSTAINABLE DEV | 0 | 0 |
| 4 |  | 0.00 | *WIK | 2009 | DIK MUT | 0 | 0 |
| 4 |  | 0.00 | **PacersFoundation | 2008 | OUR REACH | 0 | 0 |
| 4 |  | 0.00 | *AM ASS FUND RAIS | 2006 | CHAR GIV RIS 6 PERC | 0 | 0 |
| 5 |  | 0.06 | Bason T | 2015 | SPORT BUS MANAG | 5 | 218 |
| 5 |  | 0.03 | Bjarsholm D | 2017 | J SPORT MANAGE | 31 | 191 |
| 5 |  | 0.03 | Levermore R | 2015 | CORP GOV-INT J BUS S | 15 | 249 |
| 5 |  | 0.01 | Zeimers G | 2019 | EUR SPORT MANAG Q | 19 | 80 |
| 5 |  | 0.01 | Brown JA | 2018 | J BUS ETHICS | 147 | 721 |
| 5 |  | 0.01 | Jensen JA | 2017 | J SPORT MANAGE | 31 | 401 |
| 5 |  | 0.01 | Taks M | 2015 | EUR SPORT MANAG Q | 15 | 1 |
| 5 |  | 0.00 | Woisetschlager DM | 2017 | J MARKETING | 81 | 121 |
| 5 |  | 0.00 | Kim Y | 2015 | J SPORT MANAGE | 29 | 408 |
| 5 |  | 0.00 | Jones P | 2017 | INT J ENTREP INNOV | 18 | 219 |
| 5 |  | 0.00 | Miragaia DAM | 2017 | INT J SPORT POLICY P | 9 | 613 |
| 5 |  | 0.00 | Pappu R | 2014 | J ACAD MARKET SCI | 42 | 490 |
| 5 |  | 0.00 | Bergkvist Lars | 2016 | AMS REV | 6 | 157 |
| 5 |  | 0.00 | Djaballah M | 2017 | SPORT MANAG REV | 20 | 211 |
| 5 |  | 0.00 | Dos Santos MA | 2018 | INT J SPORT MARK SPO | 19 | 25 |
| 5 |  | 0.00 | Peachey JW | 2015 | J SPORT MANAGE | 29 | 570 |
| 5 |  | 0.00 | Floter T | 2016 | SPORT MANAG REV | 19 | 146 |
| 5 |  | 0.00 | Hammerschmidt J | 2020 | INT ENTREP MANAG J | 16 | 839 |
| 5 |  | 0.00 | Hemme F | 2017 | SPORT MANAG REV | 20 | 92 |
| 5 |  | 0.00 | Plewa C | 2016 | EUR J MARKETING | 50 | 796 |
| 5 |  | 0.00 | Armbrecht J | 2014 | TOURISM MANAGE | 42 | 141 |
| 5 |  | 0.00 | Inoue Y | 2017 | J BUS RES | 75 | 46 |
| 5 |  | 0.00 | Engelen A | 2015 | J MANAGE | 41 | 1069 |
| 5 |  | 0.00 | Martinez JB | 2016 | EUR J MANAG BUS ECON | 25 | 8 |
| 5 |  | 0.00 | Henseler J | 2015 | J ACAD MARKET SCI | 43 | 115 |
| 5 |  | 0.00 | Escamilla-Fajardo P | 2018 | J PHYSICAL EDUC AND SPORT | 18 | 1306 |
| 5 |  | 0.00 | Devlin M | 2018 | INT J SPORT MARK SPO | 19 | 58 |
| 5 |  | 0.00 | Smith B | 2018 | QUAL RES SPORT EXERC | 10 | 137 |
| 5 |  | 0.00 | Gonzalez-Serrano MH | 2020 | SPORT SOC | 23 | 296 |
| 5 |  | 0.00 | Vance L | 2016 | MEAS BUS EXCELL | 20 | 1 |
| 5 |  | 0.00 | Batty RJ | 2016 | J SPORT SOC ISSUES | 40 | 545 |
| 5 |  | 0.00 | Cornwell TB | 2020 | J ACAD MARKET SCI | 48 | 607 |
| 5 |  | 0.00 | Beschorner T | 2017 | J BUS ETHICS | 143 | 635 |
| 5 |  | 0.00 | Braun V | 2016 | ROUT INT HANDB | 0 | 191 |
| 5 |  | 0.00 | Preuss H | 2015 | LEISURE STUD | 34 | 643 |
| 5 |  | 0.00 | Deng XM | 2017 | J BUS ETHICS | 142 | 515 |
| 5 |  | 0.00 | Funahashi H | 2015 | MANAG SPORT LEIS | 20 | 77 |
| 6 |  | 0.18 | Alexandris K | 2012 | EUR SPORT MANAG Q | 12 | 65 |
| 6 |  | 0.17 | Lock D | 2012 | J SPORT MANAGE | 26 | 283 |
| 6 |  | 0.17 | Perez A | 2013 | EUR J MARKETING | 47 | 218 |
| 6 |  | 0.15 | Mazodier M | 2013 | J ACAD MARKET SCI | 41 | 586 |
| 6 |  | 0.15 | Norman M | 2012 | SOCIOL SPORT J | 29 | 306 |
| 6 |  | 0.06 | Hamil S | 2011 | EUR SPORT MANAG Q | 11 | 143 |
| 6 |  | 0.02 | Lock D | 2014 | J SPORT MANAGE | 28 | 119 |
| 6 |  | 0.02 | LEE J | 2011 | SPORT MARKET Q | 20 | 157 |
| 6 |  | 0.01 | Walker M | 2013 | J BUS ETHICS | 116 | 341 |
| 6 |  | 0.01 | Carrillat FA | 2012 | EUR J MARKETING | 46 | 562 |
| 6 |  | 0.00 | Beaton AA | 2011 | SPORT MANAG REV | 14 | 126 |
| 6 |  | 0.00 | Uhrich S | 2014 | J BUS RES | 67 | 2023 |
| 6 |  | 0.00 | Inoue Y | 2013 | SPORT MANAG REV | 16 | 314 |
| 6 |  | 0.00 | Hayes A F | 2013 | INTRO MEDIATION MODE | 0 | 0 |
| 6 |  | 0.00 | Pharr J R | 2012 | SPORT MARKET Q | 15 | 91 |
| 6 |  | 0.00 | Roy D P | 2011 | INT J SPORT MANAG AND MKT | 10 | 21 |
| 6 |  | 0.00 | Lee J | 2013 | SPORT MANAG REV | 16 | 161 |
| 6 |  | 0.00 | Kunkel T | 2013 | J SPORT MANAGE | 27 | 177 |
| 6 |  | 0.00 | Pielke R | 2013 | SPORT MANAG REV | 16 | 255 |
| 6 |  | 0.00 | Bigne E | 2012 | EUR J MARKETING | 46 | 575 |
| 6 |  | 0.00 | Peloza J | 2011 | J ACAD MARKET SCI | 39 | 117 |
| 6 |  | 0.00 | Walters G | 2009 | J CORPORATE CITIZENS | 35 | 81 |
| 6 |  | 0.00 | Walsh G | 2013 | J BUS RES | 66 | 989 |
| 6 |  | 0.00 | Filo K | 2011 | J LEISURE RES | 43 | 491 |
| 6 |  | 0.00 | Chien PM | 2011 | J BUS RES | 64 | 142 |
| 6 |  | 0.00 | Hallmann K | 2013 | SPORT MANAG REV | 16 | 226 |
| 6 |  | 0.00 | Geeraert A | 2014 | INT J SPORT POLICY P | 6 | 281 |
| 6 |  | 0.00 | Lacey R | 2015 | J ACAD MARKET SCI | 43 | 315 |
| 6 |  | 0.00 | Groza MD | 2011 | J BUS ETHICS | 102 | 639 |
| 6 |  | 0.00 | Kunkel T | 2016 | J SPORT MANAGE | 30 | 117 |
| 6 |  | 0.00 | **NationalCouncilforVoluntaryOrganisations | 2009 | STAT VOL SECT REC TR | 0 | 0 |
| 6 |  | 0.00 | Alcock P | 2011 | VOLUNTAS | 22 | 450 |
| 6 |  | 0.00 | **NationalBasketballAssociation | 2012 | READ TO ACH | 0 | 0 |
| 6 |  | 0.00 | **GivingUSAFoundation | 2011 | GIV US 2011 ANN REP | 0 | 0 |
| 6 |  | 0.00 | **National Counc for Vol Organ | 2011 | CHAR FOR Q SURV SECT | 0 | 0 |
| 6 |  | 0.00 | **TheDwightDHowardFoundation | 2010 | AB FDN | 0 | 0 |
| 7 |  | 0.15 | Hayhurst LMC | 2011 | THIRD WORLD Q | 32 | 531 |
| 7 |  | 0.12 | Durlak JA | 2011 | CHILD DEV | 82 | 405 |
| 7 |  | 0.12 | Spaaij R | 2012 | SPORT EDUC SOC | 17 | 77 |
| 7 |  | 0.11 | Hellison D | 2011 | TEACHING PERSONAL SO | 0 | 0 |
| 7 |  | 0.00 | Schulenkorf N | 2012 | J SPORT MANAGE | 26 | 379 |
| 7 |  | 0.00 | Coalter F | 2013 | SPORT DEV WHAT GAM | 0 | 0 |
| 7 |  | 0.00 | Wright PM | 2011 | MEAS PHYS EDUC EXERC | 15 | 204 |
| 7 |  | 0.00 | Pascual C | 2011 | RES Q EXERCISE SPORT | 82 | 499 |
| 7 |  | 0.00 | VanWynsberghe R | 2012 | J POLICY RES TOUR LE | 4 | 185 |
| 7 |  | 0.00 | Jeanes R | 2014 | GLOBAL SPORT FOR DEV | 0 | 134 |
| 7 |  | 0.00 | Veala AJ | 2012 | J POLICY RES TOUR LE | 4 | 155 |
| 7 |  | 0.00 | Peachey JW | 2015 | SPORT MANAG REV | 18 | 86 |
| 7 |  | 0.00 | Misener L | 2016 | J SPORT MANAGE | 30 | 329 |
| 7 |  | 0.00 | United Nations Development Programme | 2011 | HUM DEV IND HDI | 0 | 0 |
| 7 |  | 0.00 | World Bank | 2014 | HARDSH VULN PAC ISL | 0 | 0 |
| 7 |  | 0.00 | Balduck AL | 2011 | EUR SPORT MANAG Q | 11 | 91 |
| 7 |  | 0.00 | Schulenkorf N | 2011 | Event Management | 15 | 105 |
| 7 |  | 0.00 | **Int Fed Red Crossand… | 2011 | COOK ISL RED CROSS I | 0 | 0 |
| 7 |  | 0.00 | Darnell SC | 2011 | THIRD WORLD Q | 32 | 367 |
| 8 |  | 0.01 | Brammer S | 2008 | STRATEGIC MANAGE J | 29 | 1325 |
| 8 |  | 0.01 | *NAT BASK ASS | 2009 | NBA CAR | 0 | 0 |
| 8 |  | 0.01 | *EIAA | 2008 | EIAA SPORT SHIFT INT | 0 | 0 |
| 8 |  | 0.00 | Becker-Olsen KL | 2006 | J BUS RES | 59 | 46 |
| 8 |  | 0.00 | Sen S | 2006 | J ACAD MARKET SCI | 34 | 158 |
| 8 |  | 0.00 | Hair J | 2006 | MULTIVARIATE DATA AN | 0 | 0 |
| 8 |  | 0.00 | Weston R | 2006 | COUNS PSYCHOL | 34 | 719 |
| 8 |  | 0.00 | Vlachos PA | 2009 | J ACAD MARKET SCI | 37 | 170 |
| 8 |  | 0.00 | Berger IE | 2007 | CALIF MANAGE REV | 49 | 132 |
| 8 |  | 0.00 | Kline R B | 2005 | PRINCIPLES PRACTICE | 0 | 0 |
| 8 |  | 0.00 | Palmatier RW | 2009 | J MARKETING | 73 | 1 |
| 8 |  | 0.00 | Palmatier RW | 2006 | J MARKETING | 70 | 136 |
| 8 |  | 0.00 | Muthen LK | 2008 | MPLUS VERSION 5 2 CO | 0 | 0 |
| 8 |  | 0.00 | Newell P | 2007 | THIRD WORLD Q | 28 | 669 |
| 8 |  | 0.00 | Hair JF | 2005 | MULTIVARIATE DATA AN | 0 | 0 |
| 8 |  | 0.00 | Iacobucci D | 2007 | J CONSUM PSYCHOL | 17 | 139 |
| 8 |  | 0.00 | Simmons CJ | 2006 | J MARKETING | 70 | 154 |
| 8 |  | 0.00 | *IEG | 2009 | SPONS REP | 0 | 0 |
| 9 |  | 0.01 | Collins A | 2009 | TOURISM MANAGE | 30 | 828 |
| 9 |  | 0.01 | Siegel DS | 2007 | J ECON MANAGE STRAT | 16 | 773 |
| 9 |  | 0.00 | Pivato S | 2008 | BUS ETHICS | 17 | 3 |
| 9 |  | 0.00 | Aguilera RV | 2007 | ACAD MANAGE REV | 32 | 836 |
| 9 |  | 0.00 | Adema KL | 2010 | INT J HOSP MANAG | 29 | 199 |
| 9 |  | 0.00 | Ambec S | 2008 | ACAD MANAGE PERSPECT | 23 | 45 |
| 9 |  | 0.00 | **ChinaSportsLotteryAdministrationCenter | 2011 | ANN REP ACC 2010 | 0 | 0 |
| 9 |  | 0.00 | **AmericanGamingAssociation | 2009 | GAM REV CURR YEAR DA | 0 | 0 |
| 9 |  | 0.00 | **GamblingCommission | 2010 | ANN REP ACC 2009 10 | 0 | 0 |
| 10 |  | 0.01 | Walters G | 2009 | MANAGE DECIS | 47 | 51 |
| 10 |  | 0.00 | Smith V | 2009 | J MANAGE ORGAN | 15 | 97 |
| 10 |  | 0.00 | *EMAS | 2010 | CRED ENV MAN SYST EM | 0 | 0 |
| 10 |  | 0.00 | *FED INT FOOTB ASS | 2010 | FACT SHEET FIFA WORL | 0 | 0 |
| 10 |  | 0.00 | *INT OL COMM | 2010 | SPORT ENV COMM MISS | 0 | 0 |
| 10 |  | 0.00 | *EC PROJ INT ENV T | 2010 | ECOPROFIT ID | 0 | 0 |
